# Prevalence of and factors associated with astigmatism in preschool children in Wuxi City, China

**DOI:** 10.1186/s12886-022-02358-2

**Published:** 2022-04-01

**Authors:** Zhihui Yang, Zijing Lu, Yihui Shen, Ting Chu, Xubin Pan, Cun Wang, Jihong Wang

**Affiliations:** 1 Ophthalmology Department, Affiliated Hospital of Jiangnan University, No. 1000, Hefeng Road, Binhu District, Wuxi, 214100 China; 2 Nursing Department, Affiliated Hospital of Jiangnan University, No. 1000, Hefeng Road, Binhu District, Wuxi, 214100 China

**Keywords:** Astigmatism, Preschool children, Prevalence, Risk factors

## Abstract

**Purpose:**

To investigate the status of astigmatism in preschool children in Wuxi City, and explore the risk factors related to astigmatism. The risk factors related to astigmatism development as predictors can help us identify preschool children who need vision screening at an early stage to ensure good visual quality.

**Methods:**

The cross-sectional study was conducted in 10 kindergartens randomly selected in five districts of Wuxi City in November 2018. All preschool children were measured by objective refractometry under non-cycloplegic refraction. The basic information of preschool children was collected. The relevant factors of astigmatism in the questionnaire were completed by parents. Spss 26. 0 software was used for univariate and multivariate correlation analysis.

**Results:**

A total of 889 preschool children participated in the study, 864 were finally included in the study. The prevalence of astigmatism was 36.0%. The risk of astigmatism in premature children was higher than that in non-premature children (adjusted odds ratio = 1.841). The prevalence of astigmatism with parents’ astigmatism history was higher, compared with preschool children without parents’ astigmatism history (adjusted odds ratio = 2.037). When maternal age at childbirth was older (≥ 35 years old), the risk of astigmatism increased in preschool children (adjusted odds ratio = 2.181). Compared with bottle feeding, the risk of astigmatism for mixed feeding and breastfeeding reduced in preschool children. Compared with preschool children exposed to electronic screen for less than 2 h every day, preschool children exposed to electronic screen for more than 2 h had an increased risk of astigmatism (*P* = 0.004).

**Conclusion:**

The prevalence of astigmatism among preschool children in Wuxi City was high. Some risk factors such as premature birth, parents’ astigmatism history, maternal age at childbirth, feeding pattern, and electronic screen exposure time were closely related to the occurrence of astigmatism among preschool children. For preschool children with significant risk factors, their eyesight should be checked regularly to ensure their visual quality.

## Introduction

Astigmatism is a problem of vision blurring caused by refractive light failing to form a focus on the retina, which is a common refractive error, accounting for about 13% of refractive error [1, 2]. If astigmatism is not corrected timely, it will affect children’s visual quality, hinder their visual development and increase the possibility of amblyopia [3]. Astigmatism is a common refractive problem in Chinese children. In a survey of the prevalence of astigmatism among students in eastern China, it was found that the prevalence of astigmatism 1.00 diopter (D) or greater among children was 33%, the prevalence of astigmatism 1.50 D or greater was 14.2% and the prevalence of astigmatism 3.00 D or greater was 2.2% under non-cycloplegic refraction [4].

The etiology of astigmatism is unclear. The development of astigmatism may be influenced by both genetic and environmental factors [5]. It is reported that age, race, gene, extraocular muscle tension, eyelid pressure, smoking, electronic screen exposure time, and other factors may affect the occurrence of astigmatism [1, 6–9]. However, the relationhip between these factors and astigmatism cannot be observed in all studies [10]. Therefore, the influence of genetic and environmental factors on the development of astigmatism needs to be further explored.

The preschool period is a key period for children’s refractive development [11]. It is important to identify risk factors that may be associated with the development of astigmatism at this stage. The study analyzed the prevalence and types of astigmatism of preschool children and discussed the relationship between astigmatism and related factors by investigating the refractive characteristics of preschool children in Wuxi City.

## Methods

### Study design and population

In November 2018, a cross-sectional study was conducted of the prevalence of astigmatism and its related risk factors in preschool children from kindergartens in Wuxi City, China. Two-stage stratified cluster sampling was used to select samples. Firstly, 2 kindergartens were randomly selected from each district of Wuxi (a total of 5 districts). Then one class was randomly selected from each grade among the selected 10 kindergartens. Exclusion criteria: Children with severe eye diseases (retinal diseases, etc.) or a history of eye surgery or eye trauma were excluded. Children with contact lenses were also excluded. Children with strabismus and amblyopia and children with spectacle were included. This research was approved by the Ethics Committee of Human Research in Affiliated Hospital of Jiangnan University and was in accordance with the Helsinki Declaration. Informed consent was obtained from all participants. If participants were under 16, the informed consent was obtained from a parent and/or legal guardian. Eye examination and questionnaire survey were conducted after explaining the study to schools, parents or guardians, and children and obtaining their informed consent. A total of 889 preschool children participated in the study, 864 were finally included in the study.

### Eye examination

All preschool children received a comprehensive eye examination. Under non-cycloplegic refraction, the optometry was measured through objective refractometry (Topcon RM-800, Tokyo, Japan). Automatic continuous measurement was set up 3 times, and the average value of three readings was recorded. If the difference between different readings of the same eye was greater than 0.5 diopters, the optometry should be measured again. Ophthalmic examiners (ophthalmologists, ophthalmic nurses, and optometrists, etc.) had been trained professionally.

### Questionnaire

The questionnaire included the basic information of children (age, gender, etc.). Risk factors included premature birth (gestational age < 37 weeks, gestational age ≥ 37 weeks), delivery mode (vaginal delivery, caesarean section), feeding patterns (breastfeeding, mixed feeding, and bottle feeding), maternal age at childbirth (< 35 years old, ≥ 35 years old), parents’ smoking history (none, one, both), parents’ astigmatism history (with or without), electronic screen exposure time (< 1 h, 1to < 2 h, ≥ 2 h), outdoor activities time from Monday to Friday (< 1 h, 1 to < 2 h, ≥ 2 h), etc.

### Definition

Spherical equivalent (SE) was equal to the spherical power plus half of the cylindrical power. In either eye, SE ≤ -0.50 D was defined as myopia, SE ≥  + 2.00 D was defined as hyperopia and cylinder power ≤ -1.00 D was defined as astigmatism. According to the axis position of astigmatism, astigmatism was divided into three types: with-the-rule (WTR) astigmatism (negative cylinder axis 180 ± 30°), against-the-rule (ATR) astigmatism (negative cylinder axis 90 ± 30°), and oblique(OBL) astigmatism in other orientations.

### Statistical analysis

Firstly, univariate analysis was used to explore the relationship between related factors and astigmatism in preschool children, and to determine the risk factors related to astigmatism. Then, the factors that were significant in the univariate analysis or were considered clinically relevant were used for binary logistic regression analysis. The multivariable logistic regression model was established by forward stepwise selection. Statistically significant variables (*P* < 0.05) were retained in the multivariable logistic regression model. Odds ratio (OR) and 95% confidence interval (CI) were calculated after adjusting the influence of confounding factors. Spss 26.0 software was used for statistical analysis, and *P* < 0.05 was considered statistically significant. Due to the high correlation between the right eye and left eye, only the right eye data were used for analysis in this study.

## Results

### General characteristics

Among 889 preschool children, 35 children (2.8%) were excluded, including 10 children who were unable to cooperate with the examination or absent during the examination, 9 children with eye diseases, 12 children with a history of eye surgery or eye trauma, and 4 children with incomplete information. No childern wore contact lens. 864 preschool children (97.2%) were finally included, including 429 girls (49.7%) and 435 boys (50.3%). The average age was 4.78 ± 0.85 years old, ranging from 3 to 6 years old. The prevalence of myopia was 1.5% (*n* = 13), hyperopia was 37.6% (*n* = 325) and astigmatism was 36.0% (*n* = 311).

### The distribution of astigmatism types

**Fig. 1:**
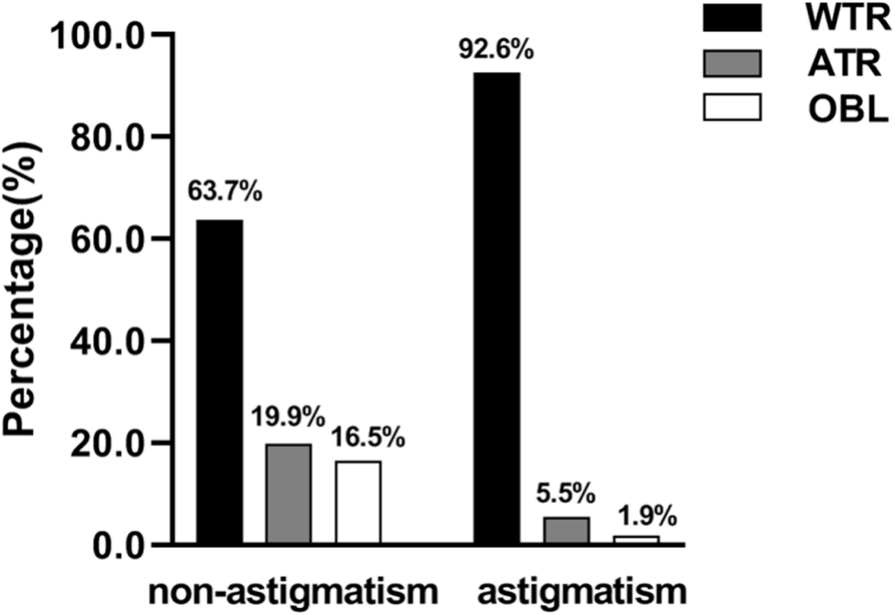
The respective percentage of WTR, ATR, OBL Figure 1 showed the respective percentage of different astigmatism types (WTR, ATR, OBL) in the astigmatism group (cylindrical power ≤ -1.00D) and non-astigmatism group (cylindrical power > -1.00D) in preschool children. The highest proportion of axial types in both groups was WTR

**Fig. 2:**
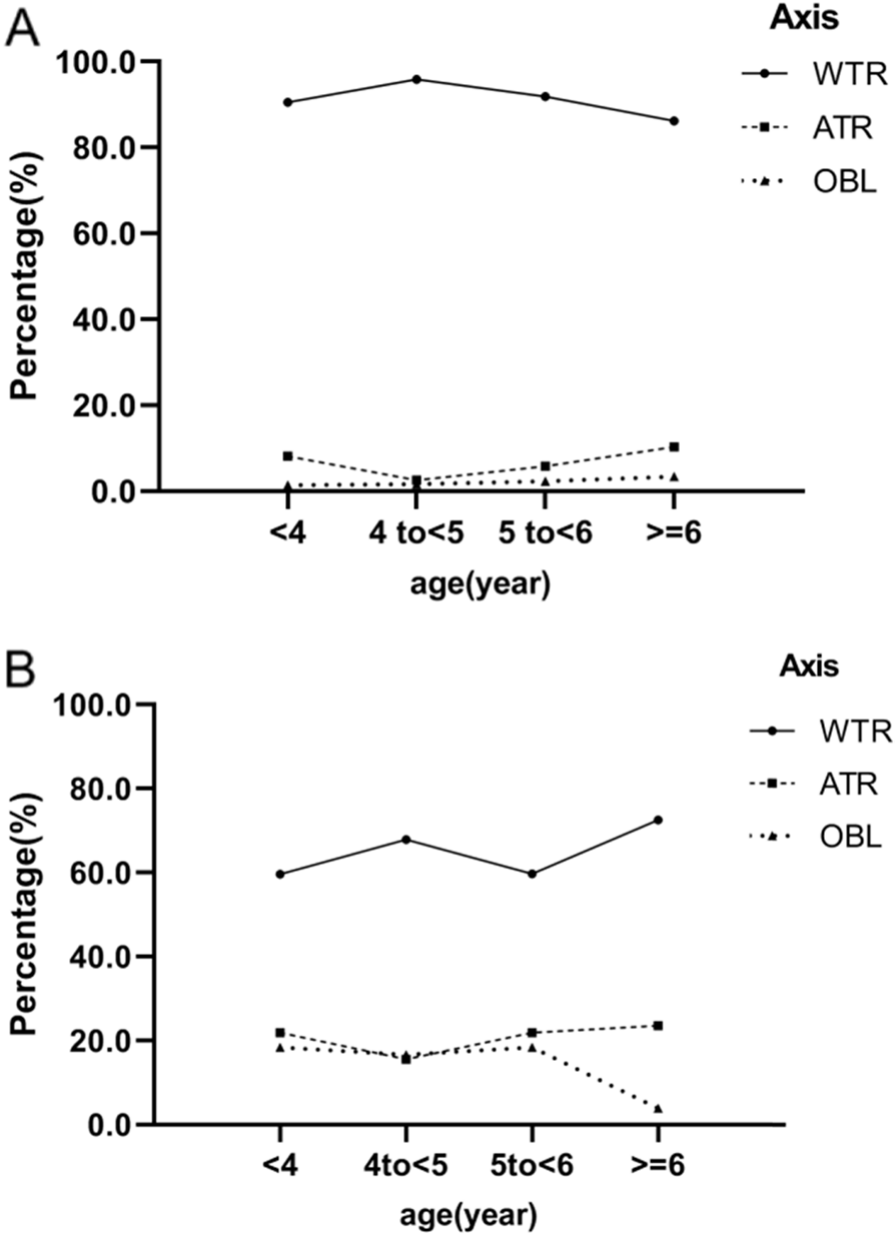
Axis changed with age. **A** Axis (WTR, ATR, OBL) changed with age in the astigmatism groups(cylindrical power ≤ -1.00D) in different age groups. **B** Axis (WTR, ATR, OBL) changed with age in non-astigmatism groups (cylindrical power > -1.00D) in different age groups

Table 1 showed the distribution of different astigmatism types (WTR, ATR, OBL) among preschool children. The proportion of different types was different. WTR accounted for the highest proportion (74.1%) among preschool children. ATR was the second, accounting for 14.7%. And finally, OBL was 11.2%. The distribution of WTR, ATR, OBL was similar between boys and girls (*p* > 0.05), but significantly different between astigmatism group (cylindrical power ≤ -1.00D) and non-astigmatism group (cylindrical power > -1.00D) (*p* < 0.001). In the astigmatism group, WTR, ATR, OBL accounted for 92.6%, 5.5%, and 1.9% respectively. In non-astigmatism group, WTR, ATR, OBL accounted for 63.7%, 19.9% and 16.5% respectively (Fig. 1). Figure 2 showed the change of different astigmatism types (WTR, ATR, OBL) with age in preschool children. It was found that WTR had the highest proportion in both the astigmatism group and non-astigmatism group in different age groups.

**Table 1:** Distribution of with-the-rule (WTR), against-the-rule (ATR), oblique (OBL) astigmatism

	Total	WTR		ATR		OBL		*P*-value	X^2^
		n	%	N	%	n	%		
All	864	640	74.1%	127	14.7%	97	11.2%		
Gender								0.288	2.492
Male	435	332	76.3%	60	13.8%	43	9.9%		
Female	429	308	71.8%	67	15.6%	54	12.6%		
Astigmatism								< 0.001	88.117
Yes	311	288	92.6%	17	5.5%	6	1.9%		
No	553	352	63.7%	110	19.9%	91	16.5%		
Age group (years)								0.029	14.022
< 4	188	135	71.8%	31	16.5%	22	11.7%		
4 to < 5	309	244	79.0%	32	10.4%	33	10.7%		
5 to < 6	287	199	69.3%	49	17.1%	39	13.6%		
≥ 6	80	62	77.5%	15	18.8%	3	3.8%

### Univariate analysis

The univariate analysis results of risk factors for astigmatism in preschool children were shown in Table 2. There was no significant difference in the prevalence of astigmatism between boys and girls, and there was no significant difference in different age groups. There was a significant correlation between premature birth and astigmatism, and the prevalence of astigmatism in premature children (48.8%) was significantly higher than that in non-premature children (34.7%) (*P* = 0.011). The prevalence of astigmatism in preschool children delivered by caesarean section (37.8%) was higher than that in preschool children delivered by vaginal delivery (34.5%), but the difference was not significant (*P* = 0.320). The risk of astigmatism in preschool children with breastfeeding history was significantly lower than that in preschool children without breastfeeding history (*P* = 0.013). Compared with preschool children without parents’ astigmatism history (astigmatism prevalence 33.9%), preschool children with parents’ astigmatism history (astigmatism prevalence 50.5%) were more likely to develop astigmatism (*p* = 0.001). Compared with preschool children without parents’ smoking history, preschool children with parents’ smoking history had a higher prevalence of astigmatism, and children whose parents both had smoking history had the highest prevalence of astigmatism, but there was no significant difference (*p* = 0.195). The risk of astigmatism increased when maternal age at childbirth was older (≥ 35 years old) (*p* = 0.037). When preschool children spent more than 2 h watching the electronic screen every day, the risk of astigmatism was significantly higher compared with children who spent less than 2 h watching the electronic screen every day (*p* = 0.005). The longer the outdoor activities time, the lower the possibility of astigmatism, but there was no significant difference (*p* = 0.278).

**Table 2:** Univariate analysis of risk factors associated with astigmatism

Factors	Total	Astigmatism		No astigmatism		*P*-value	X^2^
		n	%	N	%		
All	864	311	36.0%	553	64.0%		
Gender						0.628	0.235
Male	435	160	36.8%	275	63.2%		
female	429	151	35.2%	278	64.8%		
Age group (years)						0.069	7.088
< 4	188	74	39.4%	114	60.6%		
4 to < 5	309	122	39.5%	187	60.5%		
5 to < 6	287	86	30.0%	201	70.0%		
≥ 6	80	29	36.2%	51	63.8%		
Premature delivery						0.011*	6.428
Yes	82	40	48.8%	42	51.2%		
No	782	271	34.7%	511	65.3%		
Mode of delivery						0.320	0.988
VD	475	164	34.5%	311	65.5%		
CS	389	147	37.8%	242	62.2%		
Feeding patterns						0.013*	8.756
bottle-feeding	87	43	49.4%	44	50.6%		
mixed feeding	353	129	36.5%	224	63.5%		
breastfeeding	424	139	32.8%	285	67.2%		
Parents’ smoking history						0.195	3.276
None	491	165	33.6%	326	66.4%		
One	367	143	39.0%	224	61.0%		
Both	6	3	50.0%	3	50.0%		
Maternal age at childbirth (years)						0.037*	4.330
≥ 35	41	21	51.2%	20	48.8%		
< 35	823	290	35.2%	533	64.8%		
Parents’ astigmatism history						0.001*	11.326
Yes	109	55	50.5%	54	49.5%		
No	755	256	33.9%	499	66.1%		
Electronic screen exposure time (h/day)						0.005*	10.509
< 1 h	327	104	31.8%	223	68.2%		
1 to < 2 h	334	115	34.4%	219	65.6%		
≥ 2 h	203	92	45.3%	111	54.7%		
Outdoor activities time from Monday to Friday (h/day)						0.278	2.563
< 1 h	77	32	41.6%	45	58.4%		
1 to < 2 h	333	126	37.8%	207	62.2%		
≥ 2 h	454	153	33.7%	301	66.3%		

*VD:* vaginal delivery, *CS:* caesarean section, *: *p* < 0.05

### Binary logistic regression analysis

The significant risk factors identified in univariate analysis (premature birth, parents’ astigmatism history, maternal age at childbirth, feeding patterns, and electronic screen exposure time) were used in binary logistic regression analysis to identify independent risk factors associated with astigmatism. The final logistic model was statistically significant, χ^2^ = 39.736, *P* < 0.001, and the results were shown in Table 3. The five predictors included in the model were all statistically significant. After controlling for other confounding factors, the risk of astigmatism in premature children increased by 0.841 times (95% CI = 1.154–2.937, *P* = 0.010) compared with that in non-premature children. Children with parents’ astigmatism history had higher risk of astigmatism than children without parents’ astigmatism history (adjusted OR = 2.037, 95% CI = 1.348–3.079, *P* = 0.001). When maternal age at childbirth was older (≥ 35 years old), the risk of astigmatism of preschool children increased (adjusted OR = 2.181, 95% CI = 1.149–4.140, *p* = 0.017). There was also a relationship between astigmatism and feeding patterns. Preschool children without breastfeeding history were more likely to develop astigmatism. Compared with bottle feeding, preschool children with mixed feeding (adjusted OR = 0.572, 95% CI = 0.352–0.928) and breastfeeding (adjusted OR = 0.516, 95% CI = 0.321–0.831) reduced the risk of astigmatism.Preschool children exposed to electronic screen for more than 2 h every day had an increased risk of astigmatism (*P* = 0.004).

**Table 3:** Multivariable analysis of risk factors associated with astigmatism

Risk Factors	Total	Astigmatism		No astigmatism		OR (95% CI)	*P*-value
		n	%	N	%		
All	864	311	36.0%	553	64.0%		
Premature delivery							
No	782	271	34.7%	511	65.3%	1.0 (reference)	
Yes	82	40	48.8%	42	51.2%	1.841(1.154–2.937)	0.010
Parents’ astigmatism history							
No	755	256	33.9%	499	66.1%	1.0 (reference)	
Yes	109	55	50.5%	54	49.5%	2.037(1.348–3.079)	0.001
Maternal age at childbirth (years)							
< 35	823	290	35.2%	533	64.8%	1.0 (reference)	
≥ 35	41	21	51.2%	20	48.8%	2.181(1.149–4.140)	0.017
Feeding patterns							
bottle-feeding	87	43	49.4%	44	50.6%	1.0 (reference)	0.024
mixed feeding	353	129	36.5%	224	63.5%	0.572(0.352–0.928)	0.024
breastfeeding	424	139	32.8%	285	67.2%	0.516(0.321–0.831)	0.006
Electronic screen exposure time (h/day)							
< 1 h	327	104	31.8%	223	68.2%	1.0 (reference)	0.012
1 to < 2 h	334	115	34.4%	219	65.6%	1.129(0.811–1.573)	0.472
≥ 2 h	203	92	45.3%	111	54.7%	1.722(1.191–2.489)	0.004

*CI* confidence interval, *OR* odds ratio

## Discussion

The prevalence of astigmatism among preschool children was different in different regions of China. Previous studies have shown that the prevalence of astigmatism ranged from 4.0% to 25.4% under different definition criteria (Table 4) [11–16]. Compared with previous reports, the results of this study showed that the prevalence of astigmatism among preschool children in Wuxi City was relatively high and 36.0% of preschool children had astigmatism.

**Table 4:** Prevalence of astigmatism in different regions

Area	Study year	Sample size	Age range (years)	Definition standard of astigmatism (D)	Testing method	Cycloplegia	Prevalence (%)
HongKong [12]	2004	522	3–6	≤ -1.00 D	autorefractometer	yes	21.1%
Taiwan [13]	2010	1094	2–7	≤ -0.75 D	autorefractor	yes	25.4%
				≤ -1.00 D			13.3%
				≤ -1.50 D			4.0%
Guangxi [14]	2011	2304	3–6	≤ -1.25D	autorefraction	no	12.7%
Guangzhou [15]	2013	2480	3–6	≤ -1.50 D	autorefraction	yes	8.2%
Xuzhou [16]	2014	2255	1–6	≤ -1.00 D	retinoscopy	yes	8.8%
Shanghai [11]	2018	2851	3–6	≤ -1.00 D	autorefractor	yes	18.3%
				≤ -1.50 D			7.4%

Different studies had different reports on the axial distribution of astigmatism in preschool children. In this study, WTR had the highest proportion in both astigmatism and non-astigmatism in preschool children, which was similar to the results of previous studies [17, 18]. However, some studies believed that the type of astigmatism was predominantly ATR astigmatism [19]. Many reports showed that WTR astigmatism decreased and ATR astigmatism increased with age, but this tendency was not obvious in this study [20, 21].

Previous studies showed that delivery mode was a significant risk factor for astigmatism. Compared with preschool children delivered by vaginal delivery, preschool children delivered by selective caesarean section had an increased risk of astigmatism, which was caused by different effects of different delivery modes on the uterus, birth canal and hormones secreted [3]. This was different from our analysis results. Our univariate analysis results showed that compared with caesarean section, the prevalence of astigmatism in preschool children delivered by vaginal delivery was lower, but the difference was not significant. Therefore, the effect of delivery mode on astigmatism in preschool children still needed to be further explored.

Smoking was also an important risk factor for astigmatism. Active or passive maternal smoking during pregnancy was significantly associated with the increased risk of visual impairment in childhood, which may be related to the effect of smoking on retinal nerves and intraocular muscles [22]. It was reported that maternal smoking during pregnancy might significantly increase the prevalence of astigmatism in their children [23]. If preschool children were exposed to the smoking environment in the early stage, the degree of astigmatism also was affected, and the greater the dose of tobacco smoke exposure, the higher the risk of astigmatism [7]. However, in this study, there was no difference in the prevalence of astigmatism among preschool children with or without parents’ smoking history, which may be due to the fact that this study only investigated whether parents had smoking history, but did not clarify the specific situation of mothers’ active and passive smoking during pregnancy and children’s exposure to the smoking environment.

Univariate analysis showed that the significant risk factors of astigmatism included premature birth, parents’ astigmatism history, maternal age at childbirth, feeding pattern, electronic screen exposure time. Premature birth was an important risk factor. Compared with non-premature children, the probability of astigmatism in premature children significantly increased, which may be related to the incomplete development of the visual system in premature infants. Compared with full-term infants, the risk of abnormal visual development in premature infants significantly increased [24]. Therefore, we should pay attention to the visual development of premature children and carry out early screening. Compared with preschool children without parents’ astigmatism history, children with parents’ astigmatism history were more likely to suffer from astigmatism, which was consistent with previous results [25]. When maternal age at childbirth was older than 35 years old, it may promote the occurrence of children’s astigmatism, which was similar to the results of an analysis on risk factors of amblyopia [26]. For newborns with parents’ astigmatism history and older maternal age at childbirth, attention should be paid to their subsequent vision development.

Compared with bottle feeding, the prevalence of astigmatism in preschool children fed by breastfeeding or mixed feeding was significantly lower, which indicated that breast milk had a protective effect on preschool children’s vision development and can reduce the occurrence of astigmatism, which may be related to the effect of nutrients such as multi-chain unsaturated fatty acids in breast milk on ocular growth and development [27]. This was consistent with previous studies, which reported that there was a higher astigmatism risk in children without breastfeeding history compared with children with breastfeeding history [3]. However, some people believed that there was no significant relationship between feeding patterns and ametropia [28], so the relationship between breastfeeding and astigmatism needed to be further explored. At the same time, the ratio between breast milk and powdered milk in mixed feeding was not clear. Further studies are needed to determine the minimum percentage of visual protection provided by breast milk in mixed feeding.

Electronic screen exposure was an important factor affecting the development of vision, which may be related to the influence of lens development, increase of corneal pressure, and change of corneal shape after long-term close contact with the screen [29–31]. In a survey of preschool children in Longhua District, Shenzhen, early screen exposure was significantly correlated with the increased risk of astigmatism, and the risk of astigmatism was positively correlated with the daily screen exposure time per day and total exposure years [32]. This was consistent with our research results, which showed that the prevalence of astigmatism increased significantly when screen exposure time exceeded 2 h every day. However, this study lacked the monitoring of the duration of electronic screen exposure, which required further study and discussion.

The research showed that the prevalence of astigmatism among preschool children in Wuxi was high, which proved the necessity of carrying out large-scale refractive screening, so as to find refractive error early and correct it as soon as possible. Refractive screening needs the joint participation of several departments, including hospitals, communities, schools, and family members [33]. Since prenatal and postnatal factors are closely related to the occurrence of astigmatism, we can establish a cooperation system between ophthalmology and obstetrics. Obstetric and ophthalmologic nursing staff should focus on newborns with potential risk factors. In addition to health education for parents, early vision screening and follow-up monitoring should be carried out to ensure children’s visual quality.

Strengths of the study included the randomized selection of kindergartens and a detailed analysis of risk factors associated with astigmatism. Of course, this research had some limitations. First of all, the sample size was not large enough, and the selection of samples may be biased. Second, the risk factors assessed were not comprehensive enough, and there were still some factors that had not been assessed. Third, the refractive examination was not carried out under cycloplegic refraction, which may overestimate the prevalence of astigmatism and lead to deviation of some results. Fourth, due to the young age in preschool children, parents filled in the questionnaire instead, and parents may not be able to fully grasp the relevant information of their children, which may lead to the deviation of the results to some extent. Fifth, since this was a cross-sectional study, the temporal relationship between astigmatism and its risk factors could not be determined. Cross-sectional data cannot predict individual longitudinal changes. Population-based longitudinal studies are still needed.

## Conclusion

In conclusion, this study showed that the prevalence of astigmatism among preschool children in Wuxi City was high, and astigmatism was closely related to risk factors such as premature birth, parents’ astigmatism history, maternal age at childbirth, feeding pattern, and electronic screen exposure time. For unchangeable risk factors such as premature birth, parents’ astigmatism history, maternal age at childbirth, we focus on early vision screening so as to achieve early detection, early diagnosis, and early treatment. For modifiable risk factors such as feeding patterns and electronic screen exposure time, we can strengthen health education for parents to protect children’s eyesight.

## Data Availability

The datasets used and/or analyzed during the current study are available from the corresponding author on reasonable request.
